# An unusual presentation of adenoid cystic carcinoma of the minor salivary glands with cranial nerve palsy: a case study

**DOI:** 10.1186/1471-2407-7-157

**Published:** 2007-08-13

**Authors:** Amal Abdul-Hussein, Pierre A Morris, Tsveti Markova

**Affiliations:** 1 Department of Family Medicine, Wayne State University, Detroit, Michigan, USA

## Abstract

**Background:**

Adenoid Cystic Carcinoma (ACC) is a rare tumor entity and comprises about 1% of all malignant tumor of the oral and maxillofacial region. It is slow growing but a highly invasive cancer with a high recurrence rate. Intracranial ACC is even more infrequent and could be primary or secondary occurring either by direct invasion, hematogenous spread, or perineural spread. We report the first case of the 5^th ^and 6^th ^nerve palsy due to cavernous sinus invasion by adenoid cystic carcinoma.

**Case presentation:**

A 49-year-old African American female presented to the emergency room complaining of severe right-sided headache, photophobia, dizziness and nausea, with diplopia. The patient had a 14 year history migraine headaches, hypertension, and mild intermittent asthma. Physical examination revealed right lateral rectus muscle palsy with esotropia. There was numbness in all three divisions of the right trigeminal nerve. Motor and sensory examination of extremities was normal. An MRI of the brain/brain stem was obtained which showed a large mass in the clivus extending to involve the nasopharynx, pterygoid plate, sphenoid and right cavernous sinuses.

Biopsy showed an ACC tumor with a cribriform pattern of the minor salivary glands. The patient underwent total gross surgical resection and radiation therapy.

**Conclusion:**

This is a case of ACC of the minor salivary glands with intracranial invasion. The patient had long history of headaches which changed in character during the past year, and symptoms of acute 5^th ^and 6^th ^cranial nerve involvement. Our unique case demonstrates direct invasion of cavernous sinus and could explain the 5^th ^and 6^th ^cranial nerve involvement as histopathology revealed no perineural invasion.

## Background

Adenoid Cystic Carcinoma (ACC) is a rare tumor entity and comprises about 1% of all malignant tumor of the oral and maxillofacial region [1]. It is a slowly growing but highly invasive cancer with high recurrence rate. Lymphatic spread to local lymph nodes is rare. Hematogenous spread, however, occurs often in the course of the disease [2]. Intracranial ACC even is more rare and has been reported as 4 – 22% of ACC [3]. It could be primary or secondary which could occur either by direct invasion like in our case, hematogenous spread, or perineural spread [4,5]. Perineural spread of ACC has long been recognized. The literature revealed that the region of Gasserian ganglion to be the most common site of involvement (35.8%) [2,3,6,7], while cavernous sinus was involved in 15.1% [3-5,8,9]. Presenting signs and symptoms are related to the anatomical site of the lesion. Facial pain, parasthesia in trigeminal distribution is commonly reported reflecting the frequency of involvement of gasserian ganglia, and possibility of perineural spread along the trigeminal nerve. Involvement of cavernous sinus could be asymptomatic [3] or could present with involvement of either 3^rd^, 4^th^, 5^th^, 6^th ^and internal carotid artery [8,9]. Literature is consistent that the time between onset of neurological signs and symptoms, and the time of diagnosis range between few months to 3 years [3,6,7,10]. However, one study suggests that the duration could be several years [8].

## Case presentation

A 49-year-old African American female with a past medical history of hypertension, migraine headaches and mild intermittent asthma presented to emergency room with a severe right-sided headache for the last 3 days. The patient gave a 14-year history of migraine headaches described as a slow onset unilateral throbbing headaches without aura accompanied by nausea and lasting 2–3 days. The patient stated that her migraine attacks were decreasing in frequency and severity for the past 2–3 years until the past year when she started experiencing different kind of headache. At the time of examination, she described the headache as sharp, intermittent, non-radiating pain with sudden onset behind her right eye lasting 1–2 days. During the last 3 days the pain became more intense, reported as 10/10 on the pain scale 1 to 10, with associated photophobia, dizziness and nausea, with diplopia that was worsened with gaze to the right lateral field. Her past medical history was significant for hypertension and asthma which were stable. Her past surgical history is significant for adenoidectomy at age of 12. Her family history is significant for a sister with breast cancer, and a father who died of renal cancer. A complete review of systems was otherwise negative.

**Figure 1:**
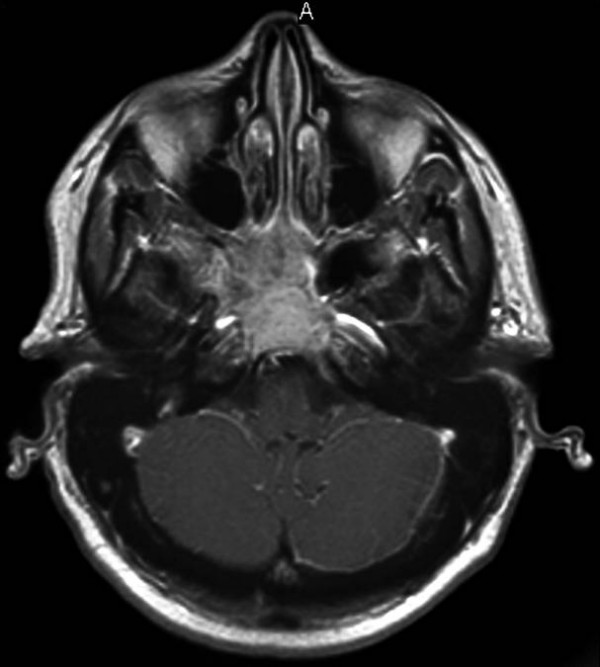
Magnetic Resonance Imaging of the Brain: a large mass in the clivus extending anteriorly to involve the nasopharynx and sphenoid sinus and posteriorly to destroy the clivus and lie anterior to the pons.

On physical examination the patient displayed right lateral rectus muscle palsy (6^th ^cranial nerve palsy) with inward deviation of her right eye. There was numbness in all three divisions of the right trigeminal nerve, suggesting involvement of the fifth cranial nerve. There was no evidence of right 3^rd^, 4^th^, 7^th ^or 8^th ^nerve involvement. Motor and sensory examination of her extremities was normal. The rest of the examination was negative. The patient was admitted to the inpatient service. Her pain was controlled with medications. All laboratory studies, including complete blood count, biochemical studies and syphilis screening were negative. A Magnetic Resonance Imaging (MRI) of the brain and brain stem revealed a large mass in the clivus extending anteriorly, involving the nasopharynx and sphenoid sinus, and posteriorly destroying the clivus laying anterior to the pons. There was destruction of the pterygoid plate and right cavernous sinus (Figure 1). Neurology consult recommended starting the patient on phenytoin and dexamethasone for seizure prophylaxis. A pituitary profile was ordered which revealed a slight elevation of prolactin level 39.1 ng/ml (normal 20 ng/ml). On day number 2 of admission, neurosurgery and otolaryngology were consulted and the patient underwent sinus endoscopy with biopsy of the right sphenoid sinus. From days 3 to 6, the patient was stable, and pain was controlled. On day 6th pathology revealed a tumor with a cribriform pattern. The neoplastic cells were monotonous and intraluminal basophilic material was noted. No perineural invasion was identified. The neoplastic cells were strongly positive for cytokertain AE1/AE3 and positive for S-100 and CD117. This histology indicated ACC of the minor salivary glands. Oncology consultant recommended resection followed by radiation. Additional work up included a thin cut computerized tomography (CT), which revealed bone invasion. Chest, abdomen and pelvis CT scans were negative for metastatic disease. The patient underwent total gross surgical resection. A postoperative CT scan showed partial resection of the tumor and the patient was scheduled for a second surgery followed by postoperative radiation.

## Discussion

Our patient's course prior to diagnosis is unusual. The patient had a history of 14-year headaches which changed in character for the past year, and symptoms of acute 6^th ^and 5^th ^cranial nerve involvement. It is difficult to determine whether the 14-year history of headache was due to slowly growing ACC with gradual invasion of clivus, nasopharnx sphenoid, and cavernous sinus. It is possible that when the patient started experiencing "different" kind of headache was a warning sign of intracranial invasion of vital structures. This is likely due to the fact that ACC is a very slowly growing tumor [2,7,8], and the size of the patient's tumor was 3.8 × 3 × 2 cm. when discovered. In our case, direct invasion of cavernous sinus could explain 6^th ^and 5^th ^cranial involvement, as histopathology revealed no perineural invasion. Based on the literature review, there are only two other cases of intracranial ACC with 6^th ^nerve involvement. One case was reported 6^th ^nerve involvement due to perineural spread [6] and second case was duo to invasion of the cavernous sinus [9].

The prognostic factors of ACC depend on tumor site, tumor stage, the presence of perineural invasion, and tumor grade. Tubular and cribriform subtypes have better prognosis than solid subtypes [2,11,12]. Many authors describe worse prognosis for tumor of the minor salivary glands, due to early local infiltration and invasion of surrounding tissue and bone [13,14] as in our reported case. The treatment of choice consists of total tumor resection [1-7,10,11]. However, there is still controversy regarding the adjuvant treatment of this tumor. Several authors recommend postoperative radiation since radiation often produces tumor regression and relieve symptoms [15-17]. Prokopakis and Kokemueller, on the other hand, doubt that postoperative radiation may influence the course of the disease [1,18]. On the other hand, chemotherapy use for ACC is controversial. Some authors report it being ineffective [9] while others had some positive response and recommend chemotherapy as palliative treatment in advance cases of ACC [19].

## Conclusion

We report a case of ACC arising from the minor salivary glands and invading the clivus nasopharynx, sphenoid, and cavernous sinuses. Controversy exists regarding the most effective treatment of ACC and there is lack of reliable information about the clinical behavior of ACC in response to treatment. Further clinical trials needed to evaluate the effectiveness of treatment on improving quality of life and survival rates. This case is a reminder that careful monitoring of headache symptoms in patients is essential and a change in characteristics should prompt further investigations.

## Competing interests

The authors declare that they have no competing interests.

## Authors' contributions

All authors, AA, PM, and TM have contributed significantly in the literature review, drafting the manuscript and revising it critically, and have given final approval for publication.

## Pre-publication history

The pre-publication history for this paper can be accessed here:


